# Coronary Artery Calcification Under Statin Therapy and Its Effect on Cardiovascular Outcomes: A Systematic Review and Meta-Analysis

**DOI:** 10.3389/fcvm.2020.600497

**Published:** 2020-12-18

**Authors:** Runmin Lai, Jianqing Ju, Qian Lin, Hao Xu

**Affiliations:** ^1^Graduate School, Beijing University of Chinese Medicine, Beijing, China; ^2^National Clinical Research Center for Chinese Medicine Cardiology, Xiyuan Hospital, China Academy of Chinese Medical Sciences, Beijing, China; ^3^Changping District Hospital of Integrated Traditional Chinese and Western Medicine, Beijing, China

**Keywords:** statins, Agatston score, coronary artery calcification, atherosclerosis cardiovascular disease, computed tomography

## Abstract

**Objective:** To compare Agatston scores between patients without statin therapy and those under standard and intensive statin therapy and to systematically review the relationship between coronary artery calcification (CAC) progression under statin therapy and cardiovascular outcomes.

**Methods:** Literature search was conducted across databases. Randomized controlled trials and observational studies that reported Agatston scores at baseline and follow-up from patients with and without statin therapy were included. A systematic review and meta-analysis was conducted.

**Results:** Seven studies were subjected to qualitative and quantitative analyses. Agatston scores in all groups were increased at follow-up. Meta-analysis of data from the included studies revealed an insignificantly lower CAC score at follow-up in the experimental groups. Subgroup analysis showed that statins slowed down CAC progression mildly but with statistical significance in population with baseline CAC score >400 in the experimental groups (*P* = 0.009). Despite that calcification progressors had worse cardiovascular outcome than did non-progressors, it appeared that baseline CAC score had more decisive effects on cardiovascular outcomes. CAC progression under statin therapy did not increase cardiovascular risk, although more supportive data are needed.

**Conclusion:** Statins do not reduce or enhance CAC as measured by Agatston score in asymptomatic populations at high risk of cardiovascular diseases, but seem to slow down CAC progression. Although our result was robust, it was restricted by small sample size and relatively short follow-up period. Further studies on the relationship between CAC progression under statin therapy and cardiovascular outcomes are needed.

**Graphical Abstract:**
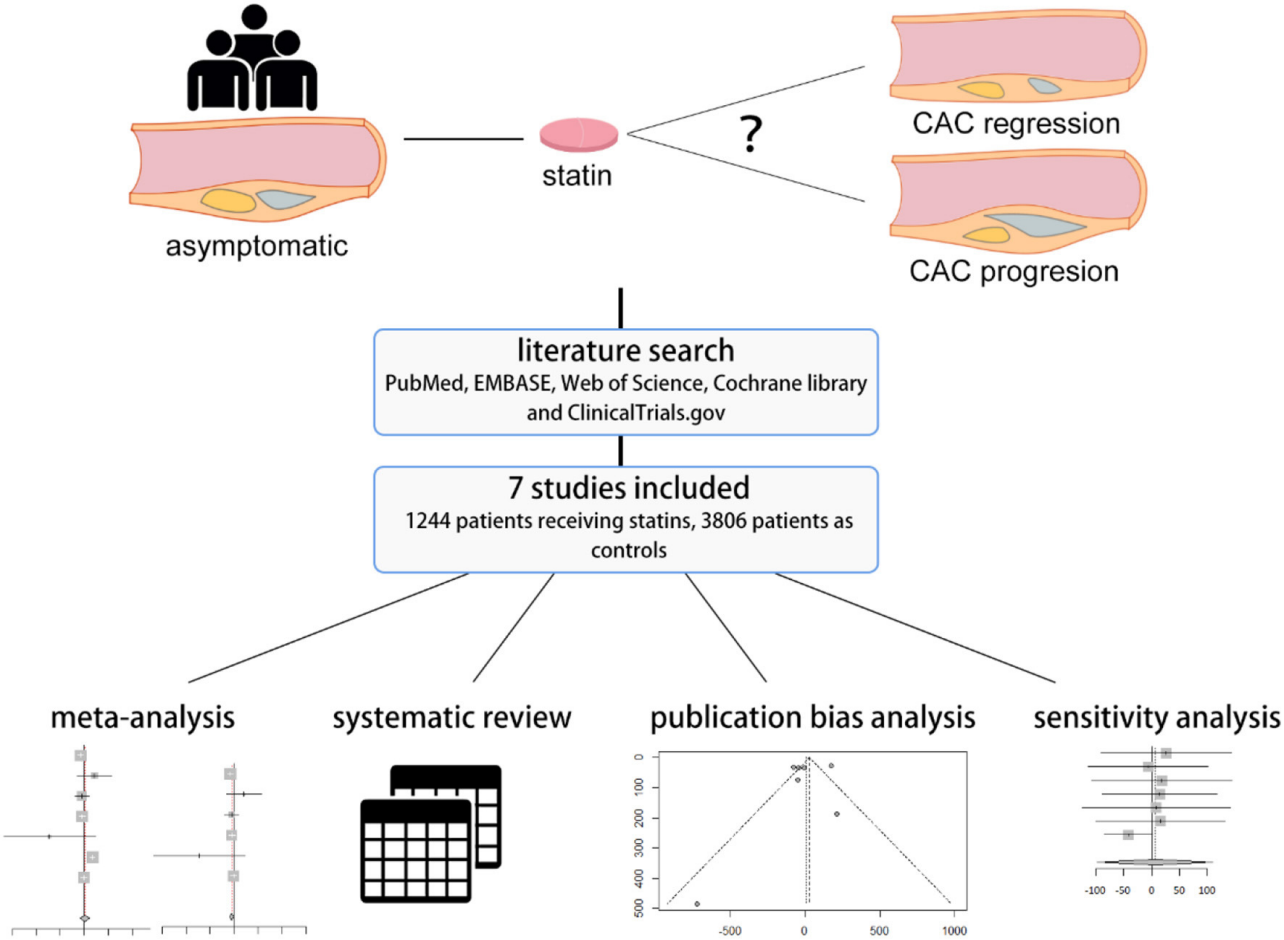
A systematic review and meta-analysis was performed to investigate coronary artery calcification change in asymptomatic population under statin therapy.

## Introduction

Calcium in coronary arteries has been used as a surrogate marker of coronary atherosclerosis since the 1940s, and its existence and progression have been correlated with higher cardiovascular risks (1). Development of various imaging techniques allows us to detect coronary calcium more accurately for cardiovascular risk assessment; nevertheless, computed tomography (CT) remains the most established and sensitive non-invasive tool to detect coronary artery calcium and to quantify it using a clinical coronary artery calcification (CAC) score (2). The Agatston scoring method of CAC remains the gold standard; it has been adopted by some major guidelines in cardiovascular risk assessment (3) and encouraged on the basis of large cohort data to be used in risk stratification for statin therapy (4).

Statins, while being effective in improving cardiovascular outcome, have also been found associated with CAC progression (5, 6). Previous meta-analyses included studies measuring coronary calcification with intravascular ultrasound (IVUS) (5) and coronary computed tomographic angiography (CCTA) (6), while the former tends to overestimate calcification due to echo shadow (1), and the latter may have difficulty in identifying and quantifying calcium in the presence of iodine in contrast media (2). To our knowledge, no previous meta-analysis has investigated the correlation between statin therapy and coronary calcification measured by Agatston score in asymptomatic populations at high risk of cardiovascular disease. Furthermore, calcification exists in the natural progression of atherosclerosis; whether statins accelerate this process or simply fail to attenuate it remains to be determined. Although it is suggested that coronary calcification enhanced by statin therapy is a marker for plaque stabilization (7), no study has systematically determined the relationship between cardiovascular outcome and coronary calcification progression under statin therapy. Therefore, we performed a systematic review and meta-analysis of CAC measured by Agatston score under statin therapy, as well as its relationship with cardiovascular outcomes.

## Methods

### Protocol and Registration

This systematic review and meta-analysis was registered on PROSPERO (CRD42020142911) and was conducted according to the Preferred Reporting Items for Systematic Review and Meta-analysis (PRISMA) (8). No patient and public are involved in the design, or conduct, or reporting of the research. All analyses were based on previous published studies; thus, no ethical approval and patient consent were required.

### Search Strategy

Literature searches were carried out in PubMed, EMBASE, Web of Science, Cochrane Library, and ClinicalTrials.gov (last search data, May 15, 2020). The following keywords in different combination were used: *statin, coronary artery, calcification* or *calcified* or *calcium*. Search categories were restricted to reviews, meta-analyses, clinical trials, letters, and conference or meeting papers. Reference lists of articles relevant to this topic were also screened. The following search strategy was used for Web of Science and modified to suit other databases:

#1 statin

#2 coronary artery

#3 calcification OR calcified OR calcium

#4 #1 AND #2 AND #3.

### Eligibility Criteria

Our study PICOS were as follows: (1) P: asymptomatic individuals at moderate to high risk of cardiovascular disease; (2) I: statin therapy of various intensity; (3) C: placebo or no use of statins (or in the case of intensive statin therapy as intervention, standard statin therapy); (4) O: CAC score as measured by Agatston method; and (5) S: both randomized controlled trials (RCTs) and observational studies. Study exclusion criteria were as follows: (1) full text not published in English; (2) results of Agatston scores arranged in quantiles; (3) CAC scores measured by other methods; (4) patients with diseases or under conditions that might seriously affect results, e.g., end-stage renal disease; (5) no discussion on the relationship between statins and CAC scores, or no comparison between experimental and control group or various statin dosage groups; and (6) study period <6 months. For different studies published under the same data source, the one with the most complete information was included.

### Data Extraction

Two authors (RM Lai, JQ Ju) independently conducted literature screens and data extractions for baseline demographic data and clinical outcomes. Any discrepancies were resolved by consensus or by a third reviewer (H Xu). Especially, Agatston scores were extracted as means with standard deviations (SDs), and those reported as medians with interquartile ranges were calculated to their means and SDs using previously described methods (9).

### Risk-of-Bias Analysis

Risk-of-bias analyses of RCTs were conducted using the Revised Cochrane risk-of-bias tool for randomized trials (RoB 2), whereas observational studies were assessed using the Newcastle–Ottawa Quality Assessment Scale (NOS).

### Statistical Analysis

The meta-analysis was performed using the *meta* package (10) in R (3.6.2). Outcomes were analyzed using a random-effects model to obtain more conservative results as we included both randomized and non-randomized studies, and heterogeneity was inevitable. Summary estimates were reported as weighted mean difference (WMD) with 95% confidence intervals (CIs). Heterogeneity among studies was tested with *Q* test and *I*^2^ statistic. A *P*-value of 0.1 was considered significant for *Q*-test, and thresholds for the interpretation of the *I*^2^ statistic were defined according to the Cochrane Handbook (11) as low heterogeneity for values from 0 to 40%, moderate heterogeneity for values from 30 to 60%, substantial heterogeneity for values from 50 to 90%, and considerable heterogeneity for values from 75 to 100%. Risk of publication bias was analyzed and presented with funnel plot. Sensitivity analysis was performed by omitting studies one at a time from the overall analysis and presented with forest plot.

## Results

### Study Selection

**Figure 1:**
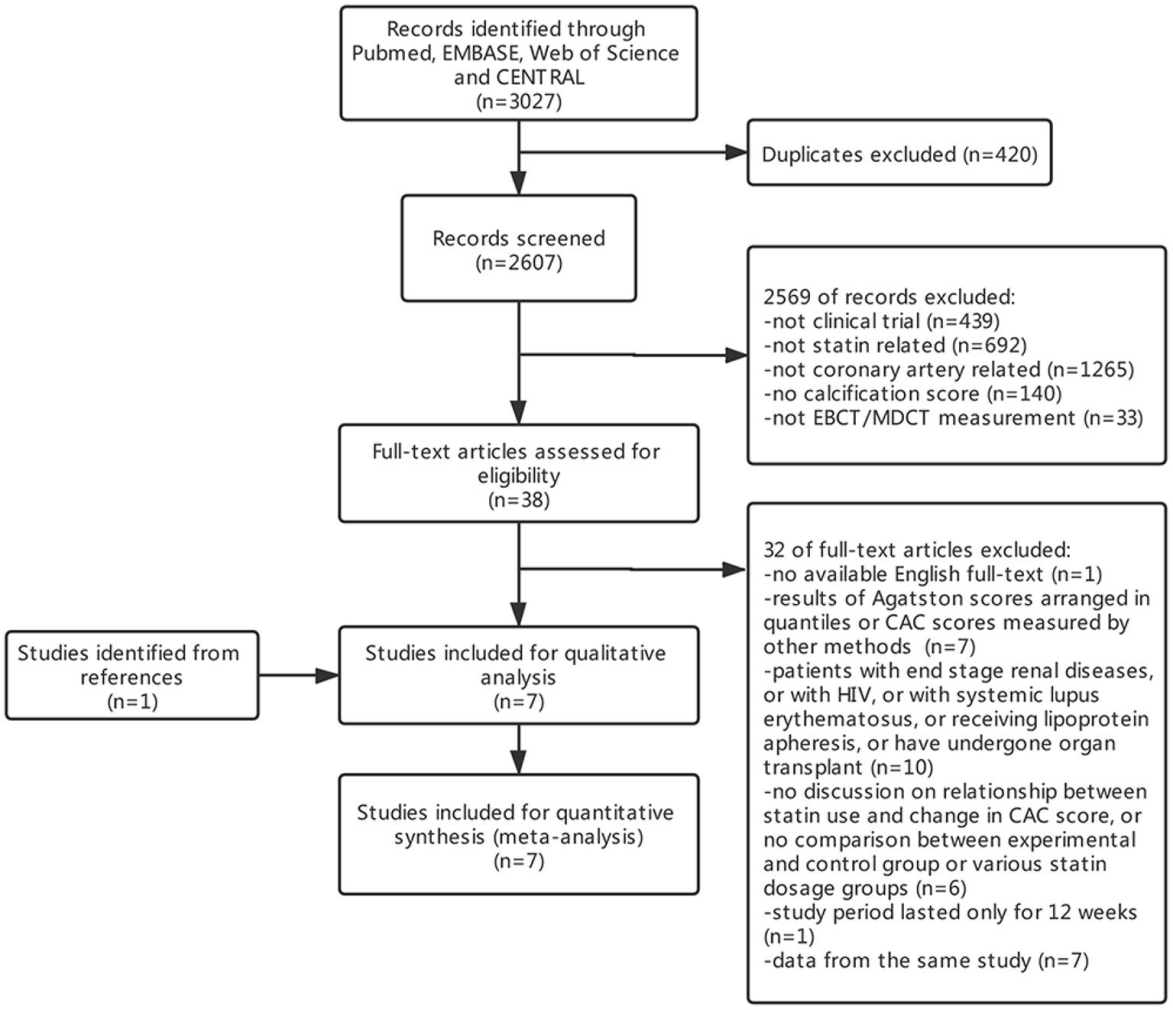
PRISMA flow chart of included studies.

A total of 3,027 initial citations were returned by searching these databases, and 2,607 items were screened by titles and abstracts after removing 420 duplicates. Thirty-eight studies were subjected to full-test review, and 32 were excluded (Figure 1). By screening reference lists of relevant articles, one study was additionally included. In all, seven studies were subjected to qualitative and quantitative analyses. During quantitative analysis, one study was excluded for its skewed distribution between experimental and control group sizes and baseline CAC scores, which exerted too much heterogeneity to the results.

### Study Characteristics

Of the seven included studies (12–18), four were RCTs (12, 16–18), and three were non-randomized observational studies (13–15). Baseline demographic and clinical characteristics of these studies are shown in Tables 1, 2. These seven studies included 1,014 patients receiving standard statin therapy, 230 patients receiving intensive statin therapy, and 3,806 patients as controls. The average age of patients ranged from 59 to 67 years. Between 46 and 98% of patients were male. The length of follow-up period ranged from 344 days to 5 years.

**Table 1:** Clinical characteristics of the included studies.

**Study**	**Study type**	**Year**	**Study arm**	***n***	**Health status**	**Statin type**	**Dosage**	**Mean follow-up time**	**Imaging method**	**Baseline CAC**	**Follow-up CAC**
Arad et al.	Randomized	2005	Treatment	417	Asymptomatic	Atorvastatin	20 mg	2 y	EBCT	528 ± 336	647 ± 438
			Control	431						563 ± 363	723 ± 483
Budoff et al.	Non-randomized	2005	Exposure	80	Asymptomatic, diabetes	NA	NA	27 mo	EBCT	797 ± 1,205	934 ± 1,238
			Control	83						511 ± 829	723 ± 1,154
Burgstahler et al.	Non-randomized	2007	Exposure	20	Asymptomatic	Atorvastatin	20 mg	488 d	MDCT	261 ± 301	293 ± 366
			Control	7						873 ± 1,101	1,017 ± 1,268
Dykun et al.	Non-randomized	2016	Exposure	230	Asymptomatic	NA	NA	5 y	EBCT	111.6 ± 201.9	238.5 ± 399.2
			Control	3,253						28.7 ± 59.5	65.3 ± 129.5
Miyoshi et al.	Randomized	2018	Standard	55	Hypercholesterolemia	Pitavastatin	2 mg	12 mo	MDCT	139.3 ± 206.3	136.7 ± 180.4
			Intensive	46			4 mg			104.0 ± 134.7	130.0 ± 153.8
Schmermund et al.	Randomized	2006	Standard	191	Dyslipidemia	Atorvastatin	10 mg	12 mo	EBCT	457 ± 704	536 ± 804
			Intensive	175			80 mg			428 ± 600	487 ± 620
Terry et al.	Randomized	2007	Treatment	30	Dyslipidemia	Simvastatin	80 mg	12 mo	MDCT	593 ± 828	645 ± 131.5
			Control	32						659 ± 725	691 ± 135.8

*CAD, coronary artery disease; EBCT, electron beam computed tomography; MDCT, multi-detector row computed tomography*.

**Table 2:** Baseline demographic of the included studies.

	**Arad et al**.	**Budoff et al**.	**Burgstahler et al**.	**Dykun et al**.	**Miyoshi et al**.	**Schmermund et al**.	**Terry et al**.
Study group	E/C	E/C	E/C	E/C	I/S	I/S	E/C
*N*	417/431	80/83	20/7	230/3,253	55/46	175/191	30/32
Age (mean ± SD)	59 ± 6/59 ± 6	64 ± 10/67 ± 10	61 ± 10	59 ± 8^c^	66 ± 10/67 ± 9	62 ± 8/61 ± 8	66 ± 5/66 ± 6
Male (%)	73/74	74^c^	46^c^	47^c^	59/53	75/74	98/85
Total cholesterol (mg/dL)	224 ± 35/227 ± 34	NA	225 ± 41/214 ± 64	NA	NA	175 ± 24/177 ± 22	198 ± 18/200 ± 19
LDL cholesterol (mg/dL)	146 ± 30/147 ± 30	NA	148 ± 7/NA	NA	NA	106 ± 22/108 ± 23	127 ± 14/129 ± 19
Hypertension (%)	38/43	52/47	21^c^	NA	83/95	83/83	NA
Diabetes mellitus (%)	9/8	100/100	8^c^	NA	28/29	13/13	NA
Current smoker (%)	12/13	14/11	6^c^	NA	20/15^b^	74/73^b^	93/90^b^
BMI (mean ± SD)	29.5 ± 5.0/29.3 ± 4.9	NA	26.7 ± 3.4^c^	NA	26 ± 5/25 ± 4	28 ± 4/27 ± 4	29 ± 5/28 ± 4
Calcium score (mean ± SD)	527 ± 336/563 ± 363^a^	796 ± 1,025/511 ± 829	261 ± 301/873 ± 1,101	111.6 ± 201.9/28.7 ± 59.5^a^	104.0 ± 134.7/139.3 ± 206.3^a^	457 ± 704/428 ± 600	593 ± 828/659 ± 725

a Data with missing SDs or reported as medians with interquartile ranges that have gone through recalculation.

b Reported as current or former smoker.

c Data only available for total study population.

Study group: E, experimental group; C, control group; I, intensive therapy; S, standard therapy.

### Risk of Bias

Among the four RCTs included, three showed a low risk of bias, one (18) showed a high risk of bias due to a high lost-to-follow-up rate, which caused CAC scores between experimental and control groups to be quite unbalanced (Supplementary Table 1). According to the NOS, all non-randomized studies were rated as high quality (Supplementary Table 2).

### CAC Assessment

Imaging processes were performed using electron beam computed tomography or multi-detector row computed tomography, with synchronized electrocardiographic triggering at 80% of the R-R interval in all but one study (13), which obtained images corresponding to 40% of the R-R interval. All studies obtained slices at 3-mm intervals but one study (18), which obtained slices at 2.5 mm intervals. Four studies made it clear that imaging interpretations were performed by blinded examiners. A calcified lesion threshold of >130 Hounsfield units was used, and the lesion score was calculated by multiplying the lesion area by a density factor derived from the maximal Hounsfield unit within this area, as described by Agatston et al. (19).

### The Effect of Statins on CAC

**Figure 2:**
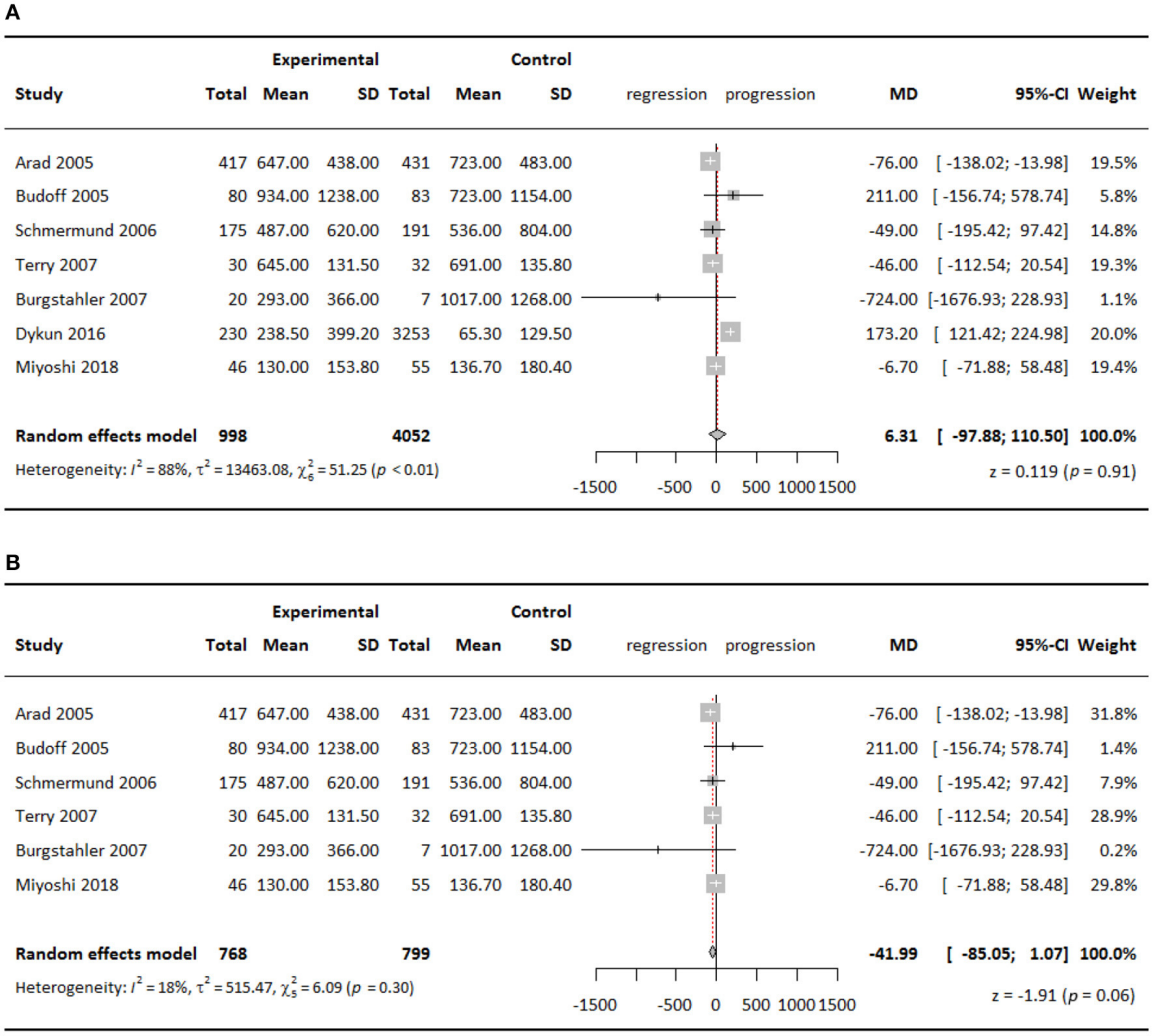
Meta-analysis of effect of statins on CAC Agatston scores comparing experimental groups to control groups, **(A)** all seven studies included; **(B)** six studies included.

Agatston scores in both experimental groups and control groups in all studies increased at follow-up compared with that at baseline. Compared with the control groups, meta-analysis of all seven studies showed higher CAC scores in the experimental groups by a WMD of 6.31 but with no significance (95% CI = 97.88–110.50; *P* = 0.91) (Figure 2A). However, including all seven studies showed substantial heterogeneity among studies (*I*^2^ = 88%), which could be largely attributed to the study of Dykun et al. (15). Because of its greatly skewed distribution of baseline CAC scores and population sizes between the experimental group and the control group, and the omission of considerable amounts of baseline demographic information, we decided to omit this study, leaving 547 patients receiving standard therapy, 221 patients receiving intensive therapy, and 799 patients in the control group in the final quantitative analysis. Heterogeneity was lowered to 18% after we omitted this study. Meta-analysis of the remaining six studies showed that CAC scores in the experimental groups were lower than that in the control groups by a WMD of 41.99 (95% CI = −85.05–1.07; *P* = 0.06) also with no statistical significance (Figure 2B), and the insignificance remained when stratifying studies according to statin therapy intensity.

**Figure 3:**
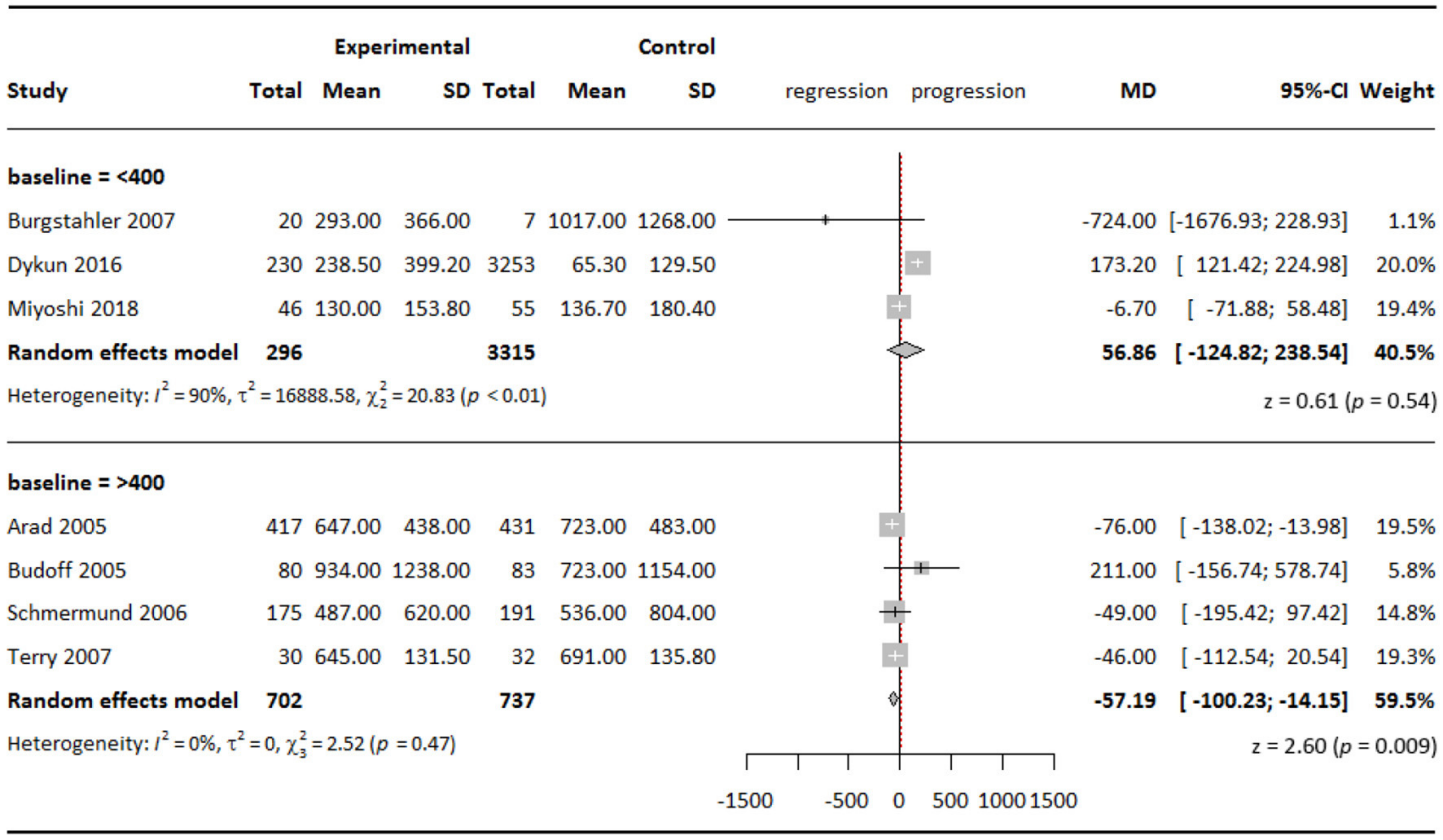
Subgroup analysis of effect of statins on CAC Agatston scores when stratified by baseline CAC score.

When stratifying studies according to baseline CAC score with a cutoff point of 400, we found that in the subgroup with baseline CAC score >400, statins slowed down CAC progression by a WMD of 57.19 (95% CI = −100.23 to −14.15; *P* = 0.009) in the experimental groups compared with the control groups (Figure 3). The effect was statistically significant, although minor.

### Publication Bias and Sensitivity Analysis

**Figure 4:**
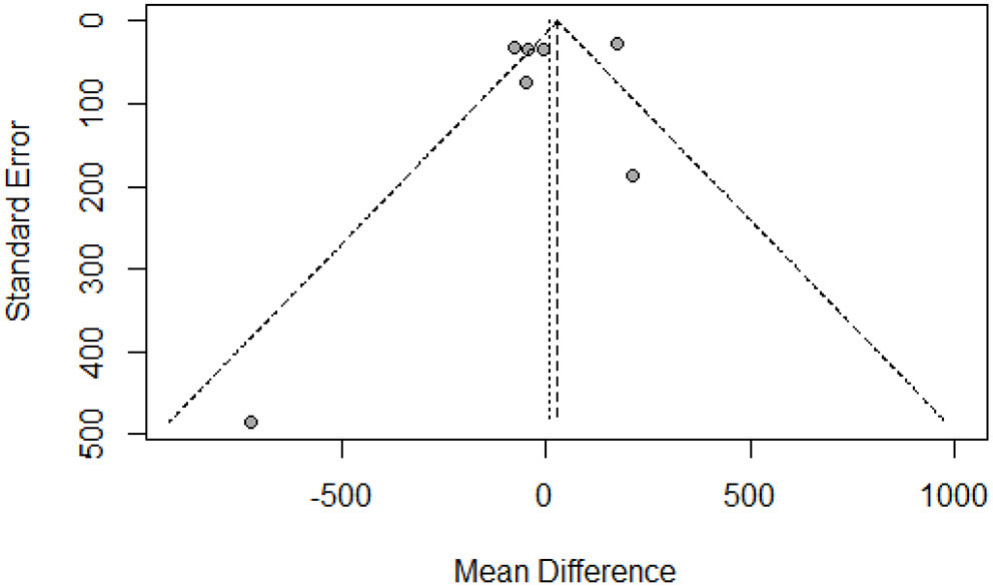
Funnel plot of the included studies.

**Figure 5:**
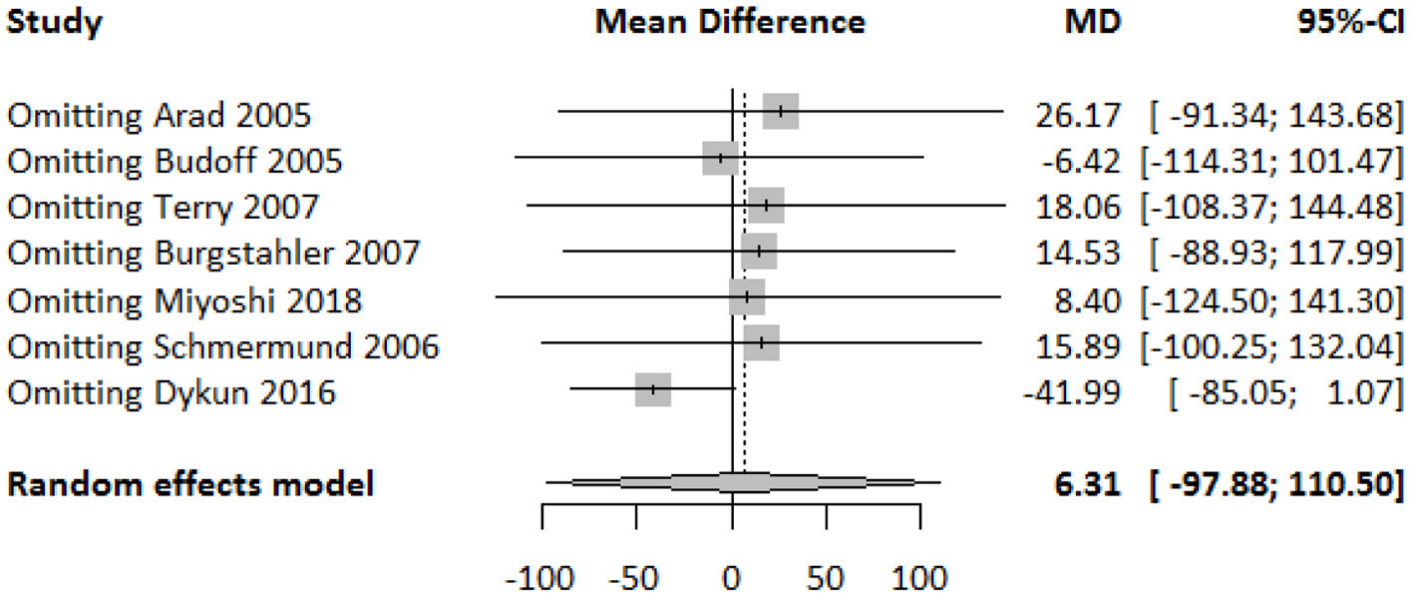
Forest plot of sensitivity analysis of the included studies.

The funnel plot showed a largely symmetrical distribution of included studies, indicating no serious publication bias (Figure 4). Sensitivity analysis showed that our result was robust. Excluding any one of the studies did not greatly affect the result (Figure 5).

### Cardiovascular Outcomes

While it is widely recognized that statin therapy improves cardiovascular outcomes, most included studies did not mention or only briefly mentioned the relationship between CAC progression under statin therapy and cardiovascular outcome. We therefore systematically reviewed and displayed cardiovascular outcomes reported by the included studies (Table 3). The overall cardiovascular outcomes between experimental group and control group were largely comparable. Dykun et al. (15) showed that statin intake enhanced CAC progression while resulting in a lower coronary event rate after performing a matched case-control analysis. On the other hand, Arad et al. (12) compared patients with and without cardiovascular events during follow-up and found out that subjects who subsequently experienced atherosclerotic cardiovascular disease events had greater absolute progression in CAC score than those who did not. However, they did not investigate cardiovascular outcomes difference between calcification progressors and non-progressors under statin therapy. In their later multivariate analysis, baseline CAC score instead of absolute change in CAC score was found to be the only significant predictor for cardiovascular events after various adjustment, while these two factors were highly positively correlated. It appeared that, compared to the progression level and rate, a lower baseline CAC score was more decisive for better cardiovascular outcomes under statin therapy.

**Table 3:** Cardiovascular outcomes related to treatment and CAC progression of the included studies.

**Study**	**Cardiovascular outcome related to statin therapy**	**Cardiovascular outcome related to CAC progression**
Arad et al.	6.9% of treatment group subjects and 9.9% of control group subjects had experienced at least one ASCVD event (*p* = 0.08). Treatment reduced all CAD events by 28% (*p* = 0.13), the sum of non-fatal MI and coronary death by 44% (*p* = 0.14), and all ASCVD events occurring more than 90 days after initiation of therapy by 33% (*p* = 0.07).	The coronary calcium score increased more from the baseline examination to the 2-year examination in subjects who subsequently experienced ASCVD events than in those who remained event-free.
Budoff et al.	NA	NA
Burgstahler et al.	No coronary adverse events occurred during follow-up.	NA
Dykun et al.	Participants with statin intake showed a tendency toward a lower coronary event rate (1.9% compared with matched subjects 2.7%, which converted into a hazard ratio of 0.74 (95% confidence interval = 0.24–2.33; *p* = 0.60).	In this population, statin intake enhanced CAC progression, mostly in the less advanced stage of atherosclerosis. However, on statin, CAC progression did not lead to increased risk of coronary events.
Miyoshi et al.	No cardiovascular events were observed during the study period.	NA
Schemermund et al.	The overall number of adverse events during the randomized treatment phase was comparable between the two groups.	NA
Terry et al.	NA	NA

### Lipid Parameters

Some of the included studies reported lipid parameters change related to statin therapy and CAC progression (Table 4). Under statin therapy, total and low-density lipoprotein (LDL) cholesterol levels in experiment groups were lowered significantly than those in the control groups in most studies. However, relationship between LDL cholesterol level and CAC progression was inconsistent among the included studies. Two (15, 16) reported that a higher LDL cholesterol level was associated with less progression in CAC, whereas the other two (17, 18) reported no significant association.

**Table 4:** Lipid parameters change related to statin therapy and CAC progression.

**Study**	**Lipid parameters change related to statin therapy**	**Lipid parameters change related to CAC progression**
Arad et al.	Differences in LDL cholesterol (*p* < 0.0001), triglycerides (0.02 > *p* ≥ 0.0001), and total cholesterol (*p* < 0.0001) between the treatment and control groups persisted throughout the study.	NA
Budoff et al.	NA	NA
Burgstahler et al.	Total cholesterol levels and LDL cholesterol levels were decreased significantly [from 225 ± 41 to 162 ± 37 mg/dL (*p* < 0.0001), and from 148 ± 7 to 88 ± 5 mg/dL (*p* < 0.001)] in the treatment group but not in the control group.	NA
Dykun et al.	NA	Statin intake in subjects with LDL cholesterol ≥115 mg/dL was associated with lower CAC progression than statin intake in subjects with LDL cholesterol levels <115 mg/dL (LDL <115 mg/dL: 56% [IQR: 17–109%], *p* = 0.003; LDL ≥115 mg/dL: 36% [IQR: 13%−63%], *p* = 0.001). After adjusting for cardiovascular risk factors, this difference was attenuated (31% [IQR: 3–77%], *p* = 0.07, and 25% [IQR: 5–50%], *p* = 0.01, respectively).
Miyoshi et al.	Intensive statin therapy significantly further lowered LDL-C level group compared with standard therapy.	In all groups, patients with higher baseline LDL-C and/or LDL-C/HDL-C ratio had significantly higher odds of non-progression of Agatston score.
Schemermund et al.	Intensive statin therapy further reduced LDL cholesterol levels by 20% (from 106 ± 22 to 87 ± 33 mg/dL), compared with standard therapy (from 108 ± 23 to 109 ± 28) (*p* < 0.05).	CAC progression showed no relationship with on treatment LDL cholesterol levels. Also, there was no relationship with any of the other on-treatment lipid parameters.
Terry et al.	Total and LDL cholesterol levels were lowered in the treatment group (from 198 ± 3 to 140 ± 3 mg/dL, and from 127 ± 2 to 74 ± 3 mg/dL), but not in the control group (*p* < 0.0001).	The magnitude of change in LDL cholesterol was not associated with a change in CAC according to the volume or Agatston score models.

## Discussion

In this systematic review and meta-analysis of statin therapy on CAC as measured by Agatston score, we demonstrated that statins failed to reduce CAC but still slowed down CAC progression in some of the asymptomatic population, instead of promoting CAC progression. Our results were partly similar to those of previous meta-analysis and studies on coronary plaque change under statin therapy measured by CT (20) and optical coherence tomography (21, 22); however, our results were inconsistent with those that measured plaque change using IVUS (5) and CCTA (6). Discrepancies between studies using different imaging methods have been mentioned previously (7). Although IVUS gives a higher resolution on plaque volume and other compositional features such as necrotic core and fibrous cap, its accuracy in measuring calcium does not necessarily exceed that of CT; more studies concerning this area are needed. While CCTA uses similar imaging techniques as CT, it introduces iodine contrast media for more morphology and composition information of plaques, which could make it difficult to precisely identify and quantify CAC (2).

The Agatston score was reported by Agatston et al. in the 1990s and has become the most commonly used method for quantifying CAC measured by various CT scanners, including electron beam, multi-detector row, and dual-source CT and CCTA (23, 24). It considers both calcium volume and density and requires only simple calculation, thereby suiting the needs of clinical practice. Despite wide application, the Agatston score has been questioned regarding its interscan reproducibility. The score could vary with higher heart rates, motion artifacts, equipment from different vendors, and software platforms (25). Other methods for calcium scoring have been developed, including the volume score and the mass score; however, none were found to be preferable to another in terms of reproducibility of results from consecutive scans in a patient (26).

We chose not to use change in CAC score from baseline to follow-up as did other meta-analyses (5, 6, 20); rather, we used specific CAC score at follow-up in statistical analysis because analyzing change scores does not control for baseline imbalance due to regression to the mean (27). Furthermore, it has been suggested that a comparable result using either absolute difference or relative difference between CAC score at follow-up and CAC score at baseline might not be achieved because of non-linear progression pattern of CAC score (23, 28). We noted that Puri et al. (5) in their meta-analysis managed to obtain a more comparable result between groups by applying a propensity score weighting method and adjusting for various covariates. However, the patients included in their meta-analysis had angiographically confirmed coronary artery disease and therefore had clinical indications for IVUS examination. A more active atherosclerotic status and later stage of plaque progression (7) could be one reason for the significant coronary calcification progression in their treatment group but not in ours. Calcification during atherosclerosis is closely associated with both progression stage and healing stage of inflammation (29), and previous studies have shown that the effect of statins on reducing inflammation was more pronounced within advanced coronary lesions as measured using ^18^F-fluorodeoxyglucose positron emission tomographic/computed tomographic imaging (30). Another reason that we did not see an enhanced CAC score due to statin therapy could be the relatively short follow-up periods of the included studies. In asymptomatic populations who have milder atherosclerosis than those with confirmed CAD, it could take longer for calcification to progress.

Interestingly, we even observed a slowdown effect from statin therapy on CAC progression in the subgroup with baseline CAC score >400, which could be a revelation that statins affect CAC differently in a less atherosclerotic but still high-risk plaque environment. Mitchell et al. (31) have previously proven that there existed a threshold of CAC score above which asymptomatic patients would benefit more from statin therapy, and they determined the threshold as 100 through a large-scale cohort analysis. In our case, the cutoff point was chosen according to distribution of baseline CAC scores across studies. More differences between various baseline CAC score intervals might be observed within a larger sample.

The relationship between CAC progression under statin therapy and cardiovascular outcome could be confusing and conflicting. Some suggested that calcification progression is a sign of micro, fragmented calcium evolving into macro, sheet calcium, which stabilizes plaques (7); other studies indicated that, in patients receiving statins and other lipid-lowering agents, those with subsequent cardiovascular events had greater CAC progression (12, 32). Previous research from the Multi-Ethnic Study of Atherosclerosis showed that, while CAC volume was positively and independently associated with cardiovascular risks, CAC density was significantly inversely associated with cardiovascular risks (33). A more recent study found out that CAC > 0 compared with CAC = 0 was associated with a significantly higher risk of atherosclerotic cardiovascular disease events regardless of baseline or incident statin use or when accounting for time-varying statin use (34). Despite the tendency of plaques becoming more calcific and stable over time under statin therapy, at an early stage of atherosclerosis, CAC progression could be both a marker of plaque stability and an indication for more intensive primary prevention therapy given different backgrounds. More studies into the mechanisms and risk factors for different cardiovascular outcomes among calcification progressors are needed.

With cholesterol, especially LDL cholesterol being an important initiator and component of atherosclerotic plaques and target of statin therapy, it is reasonable to assume an association between LDL cholesterol and CAC under statin therapy. Two included studies reported an inverse association, which is in tune with recent theory that statins stabilize plaques by promoting calcification while lowering LDL cholesterol (1); another two reported no association, which is consistent to previous findings from clinical data (5, 35, 36). Healy et al. (37) in a recent article demonstrated that statins, by inhibiting mevalonate synthesis, inhibited downstream cholesterol synthesis and activated downstream Rac1-IL-1β signaling axis. This activation led to a procalcific effect in animal models. However, considering the pleiotropic effects of statins, they could have promoted CAC through other more pathways.

## Limitations

Our study has some limitations, first being the limited number of included studies and small overall sample size, which could have restricted our conclusion from being applicable to a larger population; thus, our results should be interpreted with caution. Despite wide application of CT and Agatston score in clinical practice, few relevant studies have been done in recent years because of development of other more advanced imaging techniques. Some studies chose to publish data in the form of quantiles, progression percentiles, or calcium scores by other calculating methods, resulting in many being excluded from our analysis. Therefore, efforts to pool larger cohort data are needed.

Second, information concerning cardiovascular outcomes, especially cardiovascular outcomes related to CAC progression, is limited, partly due to the study population who were asymptomatic and atherosclerotic and relatively short follow-up period. In fact, not many studies to date have systematically evaluated CAC progression's implication on cardiovascular outcomes. Radford et al. (38) and Lehmann et al. (39) both reported based on large cohort data that CAC progression is associated with coronary and cardiovascular event rates, but adds only weakly to risk prediction. However, in both studies, participants under statin therapy took up only a small proportion of the cohorts.

Third, differences in baseline characteristics, length of follow-up period, study types, CT vendors, software platforms, and variability of Agatston scores also brought heterogeneity and some uncertainty to our result. However, our final result is robust with low to moderate heterogeneity. With CT remaining an important early screening method for cardiovascular risk, we believe that our study is of value for asymptomatic populations at high risk of cardiovascular diseases in general clinical practice.

## Conclusion and Perspectives

Statins do not reduce or enhance CAC as measured by Agatston scores in asymptomatic populations at high risk of cardiovascular diseases, but seem to slow down their CAC progression. Our results reveal the value of CT scan and CAC quantification in early screening and importance of early initiation of statins for this population. The relationship between calcification progression under statin therapy and different cardiovascular outcomes calls for further investigation, and effort to pool large cohorts with longer follow-up period and acquire individual patient data is needed.

## Data Availability Statement

The original contributions presented in the study are included in the article/Supplementary Materials, further inquiries can be directed to the corresponding author.

## Author Contributions

The research question was developed by RL. The study protocol was written and registered by RL and QL. The literature search and assessment of articles were conducted by RL and JJ, with HX acting as supervisor and a third assessor in case of disagreement authors. The manuscript was drafted by RL, polished by JJ, and revised by QL and HX. RL and HX would be responsible for the overall content in this article. All authors contributed to the article and approved the submitted version.

## Conflict of Interest

The authors declare that the research was conducted in the absence of any commercial or financial relationships that could be construed as a potential conflict of interest.

## Abbreviations

CAC: coronary artery calcification
CAD: coronary heart disease
CCTA: coronary computed tomographic angiography
CT: computed tomography
IVUS: intravascular ultrasound
LDL: low-density lipoprotein
MDCT: multi-detector row computed tomography
OCT: optical coherence tomography
RCT: randomized controlled trials.
