# Supplementary material for: Coronary Artery Calcification Under Statin Therapy and Its Effect on Cardiovascular Outcomes: A Systematic Review and Meta-Analysis
Source: Front Cardiovasc Med. 2020 Dec 18;7:600497. doi: 10.3389/fcvm.2020.600497 (PMC7793667; doi:10.3389/fcvm.2020.600497)
Supplement: Supplementary file 1 [file Table_1.DOCX]

Supplementary Table 1 Risk of bias of randomized trials

|  | Randomization | Deviation from intended intervention | Missing outcome data | Measurement of the outcome | Selection report | Overall |
| --- | --- | --- | --- | --- | --- | --- |
| Arad et al | Low | Low | Low | Low | Low | Low |
| Miyoshi et al | Low | Low | Low | Low | Low | Low |
| Schmermund et al | Low | Low | Low | Low | Low | Low |
| Terry et al | Low | Low | Some concern | Some concern | Low | High |

Supplementary Table 2 Risk of bias of nonrandomized trials

| Study | Selection | | | | Comparability | Outcome | | | Score |
| --- | --- | --- | --- | --- | --- | --- | --- | --- | --- |
|  | Representativeness of the exposed cohort | Selection of the non exposed cohort | Ascertainment of exposure | Demonstration that outcome of interest was not present at start of study | Controls for important/additional factors | Assessment of outcome | Follow-up long enough for outcomes to occur | Adequacy of follow up of cohorts |  |
| Budoff et al | ★ | ★ | ★ | ★ | - | ★ | ★ | ★ | 7 |
| Dykun et al | ★ | ★ | ★ | - | - | ★ | ★ | ★ | 6 |
| Burgstahler et al | - | ★ | ★ | ★ | - | ★ | ★ | - | 5 |
